# Lysine and novel hydroxylysine lipids in soil bacteria: amino acid membrane lipid response to temperature and pH in *Pseudopedobacter saltans*

**DOI:** 10.3389/fmicb.2015.00637

**Published:** 2015-06-29

**Authors:** Eli K. Moore, Ellen C. Hopmans, W. Irene C. Rijpstra, Irene Sánchez-Andrea, Laura Villanueva, Hans Wienk, Frans Schoutsen, Alfons J. M. Stams, Jaap S. Sinninghe Damsté

**Affiliations:** ^1^Department of Marine Organic Biogeochemistry, Royal Netherlands Institute for Sea ResearchTexel, Netherlands; ^2^Laboratory of Microbiology, Wageningen UniversityWageningen, Netherlands; ^3^NMR Spectroscopy Research Group, Bijvoet Center for Biomolecular Research, Utrecht UniversityUtrecht, Netherlands; ^4^Thermo Fisher ScientificBreda, Netherlands; ^5^Faculty of Geosciences, Utrecht UniversityUtrecht, Netherlands

**Keywords:** lysine lipid, hydroxylysine lipid, *Pseudopedobacter saltans*, *Flavobacterium johnsoniae*, stress response, soil bacteria

## Abstract

Microbial decomposition of organic matter is an essential process in the global carbon cycle. The soil bacteria *Pseudopedobacter saltans* and *Flavobacterium johnsoniae* are both able to degrade complex organic molecules, but it is not fully known how their membrane structures are adapted to their environmental niche. The membrane lipids of these species were extracted and analyzed using high performance liquid chromatography-electrospray ionization/ion trap/mass spectrometry (HPLC-ESI/IT/MS) and high resolution accurate mass/mass spectrometry (HRAM/MS). Abundant unknown intact polar lipids (IPLs) from *P. saltans* were isolated and further characterized using amino acid analysis and two dimensional nuclear magnetic resonance (NMR) spectroscopy. Ornithine IPLs (OLs) with variable (hydroxy) fatty acid composition were observed in both bacterial species. Lysine-containing IPLs (LLs) were also detected in both species and were characterized here for the first time using HPLC-MS. Novel LLs containing hydroxy fatty acids and novel hydroxylysine lipids with variable (hydroxy) fatty acid composition were identified in *P. saltans*. The confirmation of OL and LL formation in *F. johnsoniae* and *P. saltans* and the presence of *Ols*F putative homologs in *P. saltans* suggest the *Ols*F gene coding protein is possibly involved in OL and LL biosynthesis in both species, however, potential pathways of OL and LL hydroxylation in *P. saltans* are still undetermined. Triplicate cultures of *P. saltans* were grown at three temperature/pH combinations: 30°C/pH 7, 15°C/pH 7, and 15°C/pH 9. The fractional abundance of total amino acid containing IPLs containing hydroxylated fatty acids was significantly higher at higher temperature, and the fractional abundance of lysine-containing IPLs was significantly higher at lower temperature and higher pH. These results suggest that these amino acid-containing IPLs, including the novel hydroxylysine lipids, could be involved in temperature and pH stress response of soil bacteria.

## Introduction

**Figure 1:**
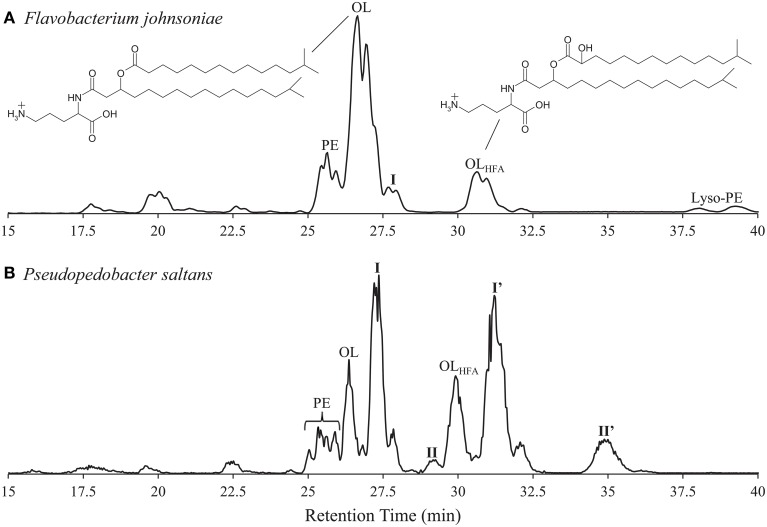
**High pressure liquid chromatography-electrospray/ion trap/mass spectrometry (HPLC-ESI/IT/MS) base peak chromatograms of (A)** ***Flavobacterium johnsoniae*** **(low abundance glycine lipids at retention time 6.5 min not shown; ornithine lipid structure included with variable fatty acid hydroxy group), and (B)** ***Pseudopedobacter saltans*** **lipid extracts**. PE, phosphatidylethanolamine; OL, ornithine lipid; OL_HFA_, Ornithine lipid with hydroxylated fatty acid; I, I', II, II”, unknown intact polar lipids. Chromatographic separation was performed on a Lichrosphere diol column (250 mm by 2.1 mm; 5-μm particles; Grace Alltech Associates Inc.). Elution was achieved with hexane–2-propanol–formic acid–14.8 M aqueous NH_3_ (79:20:0.12:0.04, v/v/v/v) **(A)** and 2-propanol–water–formic acid–14.8 M aqueous NH_3_ (88:10:0.12:0.04, v/v/v/v) **(B)** starting at 10% B, followed by a linear increase to 30% B in 10 min, followed by a 20-min hold and a further increase to 65% B at 45 min. The flow rate was 0.2 ml min^−1^, and the total run time was 60 min, followed by a 20 min re-equilibration period.

Intact polar lipids (IPLs) are useful biomarker molecules because their structures can be specific to microbial taxa or environmental conditions (Sturt et al., 2004; Schubotz et al., 2009). For example, marine phytoplankton from multiple regions of the ocean produce non-phosphorus containing lipids in response to phosphorus scarcity (Van Mooy et al., 2009), which demonstrates the importance of microbial membrane modification. IPLs containing the amino acid ornithine as the polar head group (ornithine lipids, OLs) are common phosphorus-free membrane IPLs among bacteria (Figure 1A). Approximately 50% of bacterial species whose genomes have been sequenced are predicted to have the capacity to form OLs, but they have not been predicted in eukaryotes or archaea (Lopez-Lara et al., 2003; Geiger et al., 2010; Vences-Guzmán et al., 2012, 2014). In various bacteria, OL production increases under phosphorus limitation (Weissenmayer et al., 2002; Gao et al., 2004), and in other microbes the fatty acids of OLs are hydroxylated under thermal or acid stress (Taylor et al., 1998; Rojas-Jimenez et al., 2005; Vences-Guzman et al., 2011). In eight *Desulfovibrio* strains isolated from intertidal sediments of the North Sea, relative OL content was found to increase at higher growth temperature (Seidel et al., 2013). It has also been suggested that OLs are important for Gram-negative bacterial outer membrane stability due to their zwitterionic nature (Freer et al., 1996) and essential to maintain a constant level of extracytoplasmic cytochromes in *Rhodobacter capsulatus* (Aygun-Sunar et al., 2006).

The combination of bacterial specificity, OL production and modification in response to environmental conditions, along with a suggested role in membrane stability makes this class of lipids useful potential biomarker molecules for microbial populations. Besides OLs, other amino acid-containing IPLs have also been identified including glycine lipids (Kawazoe et al., 1991; Batrakov et al., 1999), lysine lipids (Tahara et al., 1976a), glutamine lipid (Zhang et al., 2009), and an ornithine-taurine linked lipid in *Gluconobacter cerinus* (Tahara et al., 1976b) using various combinations of thin layer chromatography, infrared spectrometry, gas chromatography-mass spectrometry, electrospray ionization-mass spectrometry, and ^1^H nuclear magnetic resonance (NMR) spectroscopy. None of the lipids from these studies were identified using high performance liquid chromatography/mass spectrometry (HPLC/MS) methods, which are far less laborious than the above mentioned methods, and extremely effective for identifying IPLs in complex bacterial cultures and environmental samples (Sturt et al., 2004; Schubotz et al., 2009). The LC/MS chromatographic and fragmentation behavior of OLs is well known (Hilker et al., 1978, 1979; Tomer et al., 1983; Cerny et al., 1986 Linscheid et al., 1997; Geiger et al., 2010), but not described for various other amino acid containing IPLs. The characteristic multi-stage MS fragmentation of OLs includes the sequential loss of an H_2_O molecule from the head group, a fatty acid moiety, and the βOH-fatty acid moiety, resulting in a diagnostic *m/z* 115 cyclic fragment (Hilker et al., 1978; Cerny et al., 1986; Geiger et al., 2010). This fragmentation pattern can be used as an example for identifying other amino acid-containing IPLs via LC/MS multistage fragmentation in bacteria which are important in organic matter recycling.

Microbial organic matter decomposition is an important part of the global carbon cycle with contributions to climate change through carbon cycle feedbacks (Bardgett et al., 2008; Singh et al., 2010). The bacterial genus *Pedobacter* is of great interest to understand organic matter decomposition in soils because many species within the genus are able to degrade heparin, the biomolecule with the greatest known negative charge density (Steyn et al., 1998; Liolios et al., 2011). *Pseudopedobacter saltans* (Cao et al., 2014), originally classified in the *Pedobacter* genus (Steyn et al., 1998), has a distinct taxonomic position and its genome has the highest number of heparinase coding genes (Liolios et al., 2011). As *P. saltans* degrades highly negatively charged molecules like heparin and chondroitin, this species may be involved in the degradation of complex organic molecules in the soil such as other mucopolysaccharides or humic acids. Other microbes with the ability to degrade recalcitrant biomacromolecules are important in organic matter decomposition as well. For example, *Flavobacterium johnsoniae*, formerly *Cytophaga johnsonae*, another bacterium found in soil, rapidly digests chitin and many other macromolecules (Stanier, 1947; Larkin, 1989) and has been studied extensively for its gliding motility (McBride, 2001; McBride et al., 2003; Nelson and McBride, 2006). Both *P. saltans* and *F. johnsoniae* belong to the Cytophaga-Flavobacterium-Bacteroides (CFB) group (Stanier, 1947; Larkin, 1989; Steyn et al., 1998; Liolios et al., 2011).

Despite their important role in soil organic matter decomposition, it is not known how the membrane structural components of *P. saltans* and *F. johnsoniae* adapt to their different environmental niches. OLs and glycine lipids have both been identified in *F. johnsoniae* (Pitta et al., 1989; Kawazoe et al., 1991, 1992; Okuyama and Monde, 1996), and amino lipids have been identified in *P. saltans* (Cao et al., 2014), but the full complement of IPLs have not been described for both species. Amino acid-containing IPLs may be involved in environmental stress response by these consequential species. Here, we study the IPLs, and specifically the amino acid-containing lipids, of *P. saltans* and *F. johnsoniae* by HPLC/MS with further structural characterization by NMR spectroscopy. We report the IPL content of *P. saltans*, and *F. johnsoniae* including the structural elucidation of novel amino acid-containing lipids and discuss the potential genes and environmental factors that influence their production in *P. saltans*.

## Materials and methods

### Strains and culture conditions

*F. johnsoniae* (DSM 2064^T^) and *P. saltans* (DSM 12145^T^) were obtained from the German Collection of Microorganisms and Cell Cultures (DSMZ) in Braunschweig, Germany. *F. johnsoniae* was grown in liquid CYE medium (g per liter of distilled water): Casitone, 10.0; yeast extract, 5.0; MgSO_4_, 0.1; Tris buffer, 1.2; pH 7.2; 25°C. *P. saltans* was grown in DSMZ Medium 948 (g per liter of distilled water): Lab-Lemco powder (Oxoid), 1.0; yeast extract, 2.0; peptone, 5.0; NaCl, 5.0; pH 7.2; 25°C. Additional *P. saltans* cultures were grown in triplicate at 30°C/pH 7, 15°C/pH 7, and 15°C/pH 9 in temperature controlled rooms to determine changes in IPL fractional abundance due to different environmental conditions. The temperatures and pH levels were chosen based on temperature growth range reported by Liolios et al. (2011) and initial growth experiments, in which *P. saltans* cultures did not readily grow at temperatures below 15°C or below pH 7. The pH was adjusted by addition of HCl or NaOH solutions to achieve the desired values. Biomass from 250 ml cultures was collected by centrifugation at the stationary growth phase and freeze dried for lipid extraction and further analysis.

### Lipid extraction

Lipids were extracted from freeze dried biomass of each culture by a modified Bligh & Dyer method (Rutters et al., 2002). Biomass was fully submerged and extracted three times in methanol/dichloromethane/phosphate-buffer (MeOH/DCM/P-buffer, 2/1/0.8, v/v/v) extraction solvent for 10 min in an ultrasonic bath (P-buffer: 8.7 g K_2_HPO_4_ L^−1^ bi-distilled water adjusted to pH 7-8 with 1 N HCl). Extracts were centrifuged for 2 min at 1400 g to separate DCM phase from the MeOH/P-buffer phase, and the lower DCM layer was pipetted into a separate vial. The MeOH/P-buffer layer was washed twice more with DCM, centrifuged, and the resulting DCM layers were combined with the original DCM layer. DCM was removed under a stream of nitrogen, the residue was dissolved in injection solvent (hexane:2-propanol:H_2_O, 718:271:10, v/v/v), and filtered through a 0.45 μm, 4 mm diameter True™ Regenerated Cellulose syringe filter (Grace Davison) prior to injection. Extracts were dried down and stored at −80°C until analysis.

### HPLC-MS analysis

Intact polar lipids were analyzed by high performance liquid chromatography-electrospray ionization/ion trap mass spectrometry (HPLC-ESI/IT/MS) and HPLC-high resolution accurate mass/mass spectrometry (HRAM/MS) according to Moore et al. (2013). The HPLC-ESI/IT/MS methods closely followed Sturt et al. (2004) with some modifications (Sinninghe Damsté et al., 2011). An Agilent 1200 series HPLC, with thermostatted auto-injector, was coupled to a Thermo Scientific™ LTQ XL™ linear ion trap mass spectrometer with Ion Max source and ESI probe (Thermo Fisher Scientific, Waltham, MA). Chromatographic separation was performed on a Lichrosphere diol column (250 mm by 2.1 mm; 5-μm particles; Grace Alltech Associates Inc.). Elution was achieved with hexane–2-propanol–formic acid–14.8 M aqueous NH_3_ (79:20:0.12:0.04, v/v/v/v) (A) and 2-propanol–water–formic acid–14.8 M aqueous NH_3_ (88:10:0.12:0.04, v/v/v/v) (B) starting at 10% B, followed by a linear increase to 30% B in 10 min, followed by a 20-min hold and a further increase to 65% B at 45 min. The flow rate was 0.2 ml min^−1^, and the total run time was 60 min, followed by a 20 min re-equilibration period. Lipid extracts were analyzed by scanning the mass range of *m/z* 400–2000 in positive-ion mode, followed by data-dependent, dual-stage tandem MS (MS^2^), in which the four most abundant masses in the mass spectrum were fragmented successively (normalized collision energy, 25; isolation width, 5.0; activation Q, 0.175). Each MS^2^ was followed by data-dependent, triple-stage tandem MS (MS^3^), where the base peak of the MS^2^ spectrum was fragmented under identical fragmentation conditions to those described for MS^2^. The performance of HPLC-ESI/IT/MS was monitored by regular injections of platelet-activating factor (PAF) standard (1-*O*-hexadecyl-2-acetyl-*sn*glycero-3-phosphocholine).

HPLC-HRAM/MS analysis was accomplished on a Thermo Scientific™ Dionex™ UltiMate™ 3000 series LC with thermostatted auto-injector coupled to a Thermo Scientific™ Q Exactive™ Orbitrap™ mass spectrometer. Higher-energy Collisional Dissociation (HCD) and product ion scan were used for mass fragmentation of sample masses. The chromatographic conditions and column were the same as those described above for HPLC-ESI/IT/MS. The positive-ion ESI settings were as follows: capillary temperature, 275°C; sheath gas (N_2_) pressure, 35 arbitrary units (AU); auxiliary gas (N_2_) pressure, 10 AU; spray voltage, 4.0 kV; probe heater temperature, 300°C; S-lens, 50 V. Target lipids were analyzed with a mass range of *m/z* 400–1000 (resolution, 70,000), followed by data dependent MS^2^ (resolution, 17,500), in which the five most abundant masses in the mass spectrum were fragmented successively (normalized collision energy, 35; isolation width, 1.0). IPL fractional abundances were calculated based on HPLC-ESI/IT/MS chromatogram base peak area of each lipid group. Student's *t*-tests were performed using the GraphPad *t*-test Calculator (GraphPad Software, Inc. La Jolla, CA) in order to identify statistically significant differences in the fractional abundances of IPLs under different growth conditions; *p* < 0.05 were considered statistically significant.

### Fatty acid analysis

Aliquots of Bligh and Dyer extracts of *F. johnsoniae and P. saltans* were hydrolyzed with 1.5 N HCl in MeOH by refluxing for 3 h. The hydrolysate was adjusted to pH 4 with 2 N KOH-MeOH (1:1, v/v). Water was added to give a final ratio of 1:1 H_2_O-MeOH and this mixture was extracted three times with DCM. The DCM fractions were collected and dried over sodium sulfate. The extract was methylated with diazomethane (Sinninghe Damsté et al., 2004), followed by silylation in pyridine with *N*,*O* bis(trimethylsilyl)trifluoroacetamide (BSTFA) at 60°C for 20 min. The methylated-silylated extracts were dissolved in ethyl acetate for gas chromatography (GC)-MS analysis (Sinninghe Damsté et al., 2011). GC was performed with a Hewlett-Packard gas chromatograph (HP6890) equipped with an on-column injector and a flame ionization detector. GC-MS was performed on a Finnigan Trace Ultra gas chromatograph interfaced with a Finnigan Trace DSQ mass spectrometer operated at 70 eV with a mass range of *m/z* 40–800 and a cycle time of 1.7 s (resolution, 1000).

### Target lipid isolation

Isolation of target lipids from *P. saltans* and known OL from *F. johnsoniae* lipid extracts was accomplished using an Agilent Technologies (Santa Clara, CA) 1100 series LC equipped with an auto-injector, and a fraction collector (Foxy Jr., Isco, Inc., Lincoln, NE). A first isolation was achieved on a semi-preparative LiChrospher diol column (10 × 250 mm, 5 μm; Grace Alltech Associates Inc.) according to Boumann et al. (2009) with the same gradient program as described above for HPLC-ESI/IT/MS, but at a flow rate of 3 ml min^−1^. Typical injection volume was 200 μL containing up to 2 mg material per injection. Column effluent was collected in 1 min fractions, which were screened for the presence of target lipids by flow injection analysis *cf*. Smittenberg et al. (2002) using the ESI/IT/MS (5 μl injection of each fraction, ESI source settings same as described above for HPLC-ESI/IT/MS with a scan range of *m/z* 400–2000). Fractions containing target lipids were pooled and further purified on a second LiChrospher diol column (4.6 × 250 mm, 5 μm; Grace Alltech Associates Inc.). Typical injection volumes were 65 μl containing up to 0.65 mg of material. Lipids were eluted using the identical gradient program and conditions as described above for HPLC-ESI/IT/MS at a flow rate of 1 ml min^−1^, however mobile phases A and B did not contain NH_3_ or formic acid. Column effluent was collected in 15 s fractions and screened as described above. Fractions containing target lipids were again combined and elution solvent was removed under a stream of nitrogen. Purity was assessed by HPLC-ESI/IT/MS as described above for IPL analysis. Isolated lipids were stored at −80°C prior to further analysis.

### Nuclear magnetic resonance

The purified lipids were dissolved in 99.9% CDCl_3_ at concentrations of 1.88 and 1.83 mmol ml ^−1^, and NMR experiments were performed at 298 K on a Bruker 600-MHz Avance spectrometer equipped with 5 mm TCI cryoprobe and running under TOPSPIN 2.1. One-dimensional (1D) ^1^H and ^1^H-decoupled 1D ^13^C and DEPT-135 experiments were recorded with spectral widths/offsets of 24 ppm/6 ppm, 200 ppm/100 ppm, and 200 ppm/100 ppm and with 16,384 (16k), 4096 (4k), and 4096 (4k) complex points, respectively. The 12- by 12-ppm 2D correlation spectroscopy (COSY), total correlation spectroscopy (TOCSY), and nuclear Overhauser effect spectroscopy (NOESY) experiments were performed with 512 by 200 complex points (2k by 256 for the COSY) and an offset frequency of 5 ppm. Mixing times were 60 ms and 250 ms for TOCSY and NOESY, respectively. The (^1^H, ^13^C)-heteronuclear single-quantum correlation spectroscopy (HSQC) and (^1^H, ^13^C)-heteronuclear multiple-bond correlation spectroscopy (HMBC) experiments were recorded with spectral widths/offsets of 12/5 ppm for protons, 200/100 ppm for ^13^C HSQC (512 × 60 complex points) and 150/75 ppm for the HMBC (1k × 80 complex points). All spectra were calibrated with respect to internal residually protonated CHCl_3_ at 7.24 ppm (^1^H) and 77.0 ppm (^13^C).

### Amino acid analysis

Approximately 8.4 nmol of each of the purified *P. saltans* lipids, and 40 nmol of a known ornithine containing lipid purified from *F. johnsoniae* used as a reference compound, were hydrolyzed in 0.5 ml of 6 M HCl at 110°C for 18 h to liberate the amino acid head groups from the fatty acids for amino acid analysis. Standardized methods for the analysis of the amino acid lipid hydrolysates follow Spackman et al. (1958), Commission Regulation (EC) no. 152 (2009)^1^, and Eur. Ph. Chapter 2.2.56 (2005)^2^ where they are described in detail. Added to each hydrolysate vial was 240 μl of Li-loading buffer (Li-citrate pH 2.2) and 80 μl of internal standard (IS) solution (0.5 mM norleucine in 5% 5-sulfosalicylic acid). After mixing and re-dissolving the mix was filtered using a PALL 0.2 μm, 13 mm GHP Acrodisk. Amino acid analysis (IS-method, one step calibration vs. standard solution A9906, Sigma-Aldrich) was performed using a Biochrom 30 amino acid analyzer by Ansynth Service B.V. (Roosendaal, Netherlands). Separation was performed on a weak acidic cation exchange resin stationary phase (200 × 4.6 mm column) with a mobile phase consisting of number of weak acidic Li-citrate buffers. Stepwise pH, temperature and salt concentration gradients were applied. Spectrophotometer detection occurred after post column derivatization with ninhydrin (135°C) at 570 or 440 nm.

## Results and discussion

### IPLs of *F. johnsoniae*

The IPLs of *F. johnsoniae* identified using HPLC-ESI/IT/MS analysis included abundant OLs, moderately abundant hydroxylated fatty acid OLs (OL_HFA_) and phosphatidylethanolamines (PEs), and low abundance lyso-PE (PE with one fatty acid), and glycine lipids, which make up 47, 11, 12, 2, and 1%, respectively of the total HPLC-ESI/IT/MS chromatogram base peak area (Figure 1A). OL_HFA_ were hydroxylated at the α-position of the ester linked fatty acid (Figure 1A). In addition, two low abundance IPLs with OL-like fragmentation were observed at retention times 27.7 and 28.0 min with apparent protonated molecules ([M+H]^+^) at *m/z* values 639 and 625, respectively, making up 4.6% of HPLC-ESI/IT/MS chromatogram base peak area, which we will refer to as group I. The group I IPLs displayed sequential fragmentation loss of an H_2_O molecule, a fatty acid moiety, and a βOH-fatty acid, reminiscent of the fragmentation of OLs, but resulting in MS^3^ fragment ions at *m/z* 129, 130, and 147, instead of the characteristic *m/z* 115 OL MS^3^ fragment.

**Figure 2:**
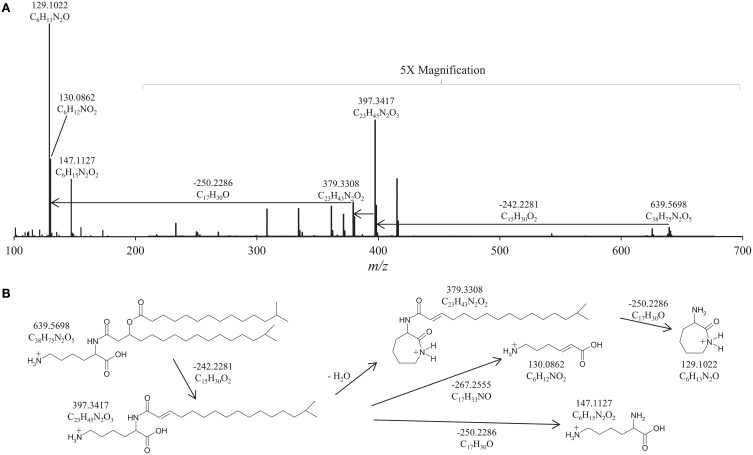
**High Resolution Accurate Mass/Mass Spectrometer (HRAM/MS) fragmentation of (A)** ***Flavobacterium johnsoniae*** **group I lipid at retention time 27.70 min; (B) proposed MS fragmentation of lysine lipids (LL) observed in** ***F. johnsoniae***. The relative abundance of the *m/z* 200-700 range is magnified 5×. The positive-ion ESI settings were as follows: capillary temperature, 275°C; sheath gas (N_2_) pressure, 35 arbitrary units (AU); auxiliary gas (N_2_) pressure, 10 AU; spray voltage, 4.0 kV; probe heater temperature, 300°C; S-lens, 50 V. Target lipids were analyzed with a mass range of *m/z* 400–1000 (resolution, 70,000), followed by data dependent MS^2^ (resolution, 17,500), in which the five most abundant masses in the mass spectrum were fragmented successively (normalized collision energy, 35; isolation width, 1.0).

To further elucidate the identity of the group I lipids we analyzed the *F. johnsoniae* lipid extract by HPLC-HRAM/MS, which revealed the elemental composition of the *m/z* 129, 130, and 147 fragmentation products of group I (Figure 2, Table 1). During fragmentation the loss of fatty acids and H_2_O from the head group likely resulted in the formation of a ring structure as described for the fragmentation of OL (Zhang et al., 2009), giving the most abundant fragmentation product of *m/z* 129.1022 with elemental composition C_6_H_13_N_2_O. The loss of the fatty acid moieties without the loss of H_2_O results in the *m/z* 147.1127 fragmentation product, which has the elemental composition (C_6_H_15_N_2_O_2_) of protonated lysine, and further loss of NH_3_ from this fragment results in the *m/z* 130.0862 fragment ion (C_6_H_12_NO_2_). The elemental composition of the *m/z* 129.1022 fragment also corresponds to protonated lysine minus H_2_O. Elemental compositions of fatty acid losses corresponded to the expected molecular formulas of regular and βOH-fatty acids. Based on this information, we propose that group I IPLs in *F. johnsoniae* are lysine-containing lipids (LLs) with different fatty acid compositions (Table 1, lysine lipid, with 15C:0 and βOH-17C:0 fatty acids shown in Figure 2). GC/MS analysis showed that the most abundant 15C:0 and βOH-17C:0 fatty acids were *iso* branched (Figure 1A) as previously reported (Pitta et al., 1989; Okuyama and Monde, 1996).

**Table 1:** **Elemental composition of amino acid containing lipid fragmentation products observed by high performance liquid chromatography-high resolution accurate mass/mass spectrometry (HPLC-HRAM/MS) in the lipid extracts of** ***Flavobacterium johnsoniae*** **and** ***Pseudopedobacter saltans***.

**Species/Lipid**	***m/z***	**RT (min)**	***m/z*, Product**	**Δmmu**	***m/z*, Product**	**Δmmu**	***m/z*, Product**	**Δmmu**
***Flavobacter johnsoniae***								
I - 15C:0, βOH-17C:0	639	27.70	147, C_6_H_15_N_2_O_2_	0.074	130, C_6_H_12_NO_2_	0.055	129, C_6_H_13_N_2_O	0.010
I - 15C:0, βOH-16C:0	625	27.95	147, C_6_H_15_N_2_O_2_	0.114	130, C_6_H_12_NO_2_	0.075	129, C_6_H_13_N_2_O	0.040
***Pseudopedobacter saltans***								
I—15C:0, βOH-17C:0	639	27.53	147, C_6_H_15_N_2_O_2_	0.006	130, C_6_H_12_NO_2_	0.095	129, C_6_H_13_N_2_O	0.100
I—15C:0, βOH-16C:0	625	27.93	147, C_6_H_15_N_2_O_2_	0.006	130, C_6_H_12_NO_2_	0.095	129, C_6_H_13_N_2_O	0.100
I—15C:0, βOH-15C:0	611	28.05	147, C_6_H_15_N_2_O_2_	0.024	130, C_6_H_12_NO_2_	0.075	129, C_6_H_13_N_2_O	0.080
I'—OH-15C:0, βOH-17C:0	655	31.73	147, C_6_H_15_N_2_O_2_	0.026	130, C_6_H_12_NO_2_	0.105	129, C_6_H_13_N_2_O	0.110
I'—OH-15C:0, βOH-16C:0	641	32.44	147, C_6_H_15_N_2_O_2_	0.006	130, C_6_H_12_NO_2_	0.095	129, C_6_H_13_N_2_O	0.100
I'—OH-15C:0, βOH-15C:0	627	32.76	147, C_6_H_15_N_2_O_2_	0.016	130, C_6_H_12_NO_2_	0.105	129, C_6_H_13_N_2_O	0.100
II—15C:0, βOH-17C:0	655	29.59	163, C_6_H_15_N_2_O_3_	0.319	145, C_6_H_13_N_2_O_2_	0.126	128, C_6_H_10_NO_2_	0.145
II'—OH-15C:0, βOH-17C:0	671	35.97	163, C_6_H_15_N_2_O_3_	0.189	145, C_6_H_13_N_2_O_2_	0.064	128, C_6_H_10_NO_2_	0.055
II'—OH-15C:0, βOH-16C:0	657	36.91	163, C_6_H_15_N_2_O_3_	0.219	145, C_6_H_13_N_2_O_2_	0.156	128, C_6_H_10_NO_2_	0.155
II'—OH-15C:0, βOH-15C:0	643	37.42	163, C_6_H_15_N_2_O_3_	0.069	145, C_6_H_13_N_2_O_2_	0.136	128, C_6_H_10_NO_2_	0.135

Retention time, RT; Accuracy, Δmmu^*^; OH, hydroxy group attached to fatty acids; Gln, glutamine; I, proposed lysine lipid (LL); I', proposed lysine lipid with hydroxylated fatty acid (LL_HFA_); I, proposed hydroxylysine lipid (HLL); II', proposed hydroxylysine lipid with hydroxylated fatty acid (HLL_HFA_).

*In general the 0.5 Δmmu (milli mass unit) range represented very high confidence molecular formula assignments and the 1.0 Δmmu range represented good confidence molecular formula assignments (Kostiainen et al., 1997; Sakayanagi et al., 2006; Calza et al., 2012).

### IPLs of *P. saltans*

HPLC-ESI/IT/MS analysis of the lipid extract of *P. saltans* showed abundant PE, OL, and OL_HFA_ IPLs (Figure 1B) making up 8, 10, and 12%, respectively, of HPLC-ESI/IT/MS chromatogram base peak area and four clusters with unknown IPLs, groups I, I', II, and II' making up 23, 28, 1.5, and 7% of HPLC-ESI/IT/MS chromatogram base peak area, respectively. OL_HFA_ were hydroxylated on the ester linked fatty acids at the α-position. *P. saltans* group I produced MS^3^ fragmentation products of *m/z* 129, 130, and 147, identical to the group I LLs identified in *F. johnsoniae*. The relative retention time of group I IPLs in *P. saltans* are also similar to the retention time of the LLs in *F. johnsoniae* (Table 1), and were therefore also identified as LLs. This was confirmed by HPLC-HRAM/MS analysis (Table 1). *P. saltans* group I' IPLs exhibited the same MS^3^ fragmentation products (*m/z* 129, 130, and 147) as group I LLs, but MS^2^ fatty acid fragmentation losses were 16 Th larger than observed for group I, indicating the IPLs in group I' are potentially hydroxylated-fatty acid versions of group I LLs (LL_HFA_). This was also confirmed by HPLC-HRAM/MS analysis (Table 1). As with OL_HFA_, group I' LL_HFA_ were hydroxylated on the ester linked fatty acid α-position. The most abundant *P. saltans* fatty acids were *iso* branched as previously observed (Steyn et al., 1998; Liolios et al., 2011), and the distribution of fatty acid chain lengths and double bond equivalents were identical between the LLs, LL_HFA_, OLs, and OL_HFA_, suggesting a biosynthetic link between these IPL classes (Table 1).

Group I LLs and group I' LL_HFA_ were isolated from the *P. saltans* extract using preparatory HPLC to confirm the lysine head group structure. Approximately 0.6 mg of both LL and LL_HFA_ were individually isolated for NMR analysis. For comparison, roughly 2.0 mg of known OL was also isolated from the *F. johnsoniae* extract. Results from the ^1^H-NMR, COSY, and ^13^C-NMR analysis of isolated LLs and LL_HFA_ (Table 2; Supplementary Materials) revealed many similarities with NMR characterization reported for OLs (Okuyama and Monde, 1996; Linscheid et al., 1997; Moore et al., 2013). The fatty acid chain chemical shifts were almost identical between LLs, LL_HFA_, and OLs, except for the OH group attached to the hydroxylated fatty acids of LL_HFA_. There were additional ^1^H and ^13^C signals for the head group of LLs and LL_HFA_ corresponding to the ε-position of the lysine head group, compared to the OL head group, in agreement with the fact that lysine has one more carbon than ornithine. The most compelling 2D-NMR evidence of a lysine head group in the proposed LLs and LL_HFA_ was observed in the TOCSY interactions among the head group positions (Table 2). The same TOCSY interactions were previously described in the structural determinations of components containing an esterified lysine moiety (Anderson et al., 1995), and peptide-bound lysine (Rabenstein et al., 1997). Interactions observed by adjacent carbon and proton atoms in the HSQC spectra, and spatially close protons in NOESY spectra, further supported the lysine head group assignment of LLs and LL_HFA_. Amino acid analysis of each of the isolated LL, LL_HFA_, and OL components confirmed the presence of a lysine head group in the LL and LL_HFA_ and an ornithine head group in the OL (Table 3).

**Table 2:**
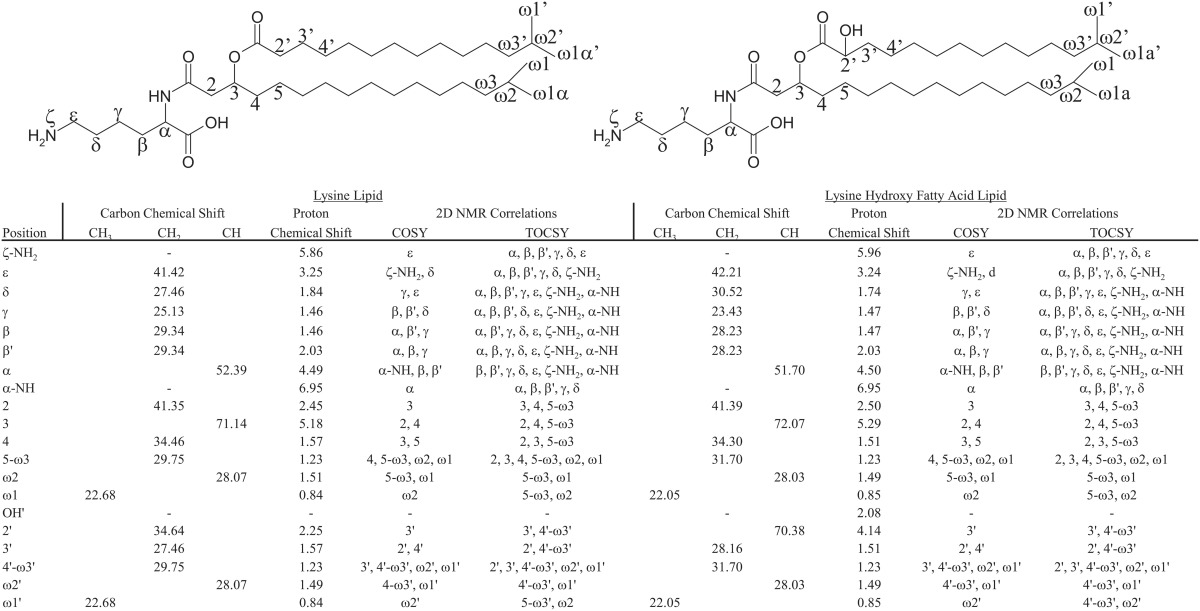
**Nuclear magnetic resonance (NMR) proton and carbon chemical shifts, and 2D NMR correlations (correlation spectroscopy: COSY; total correlation spectroscopy: TOCSY) of lysine lipid (LL) and lysine hydroxy fatty acid lipid (LL_HFA_)**.

**Table 3:** **Amino acid analysis retention times (RT) of amino acids released from ornithine lipid, proposed lysine lipid (group I, LL), proposed lysine lipid with hydroxylated fatty acid (group I', LL_HFA_), and proposed hydroxylysine lipid with hydroxylated fatty acid (group II', HLL_HFA_) hydrolysates**.

**Amino acid standard**	**Lipid head group**	**Amino acid standard RT (min)**	**Lipid hydrolysate head group RT (min)**
Ornithine	Ornithine	100.11	100.11
Lysine	I, I'	103.58	103.59, 103.61
Hydroxylysine	II'	95.99	95.63

**Figure 3:**
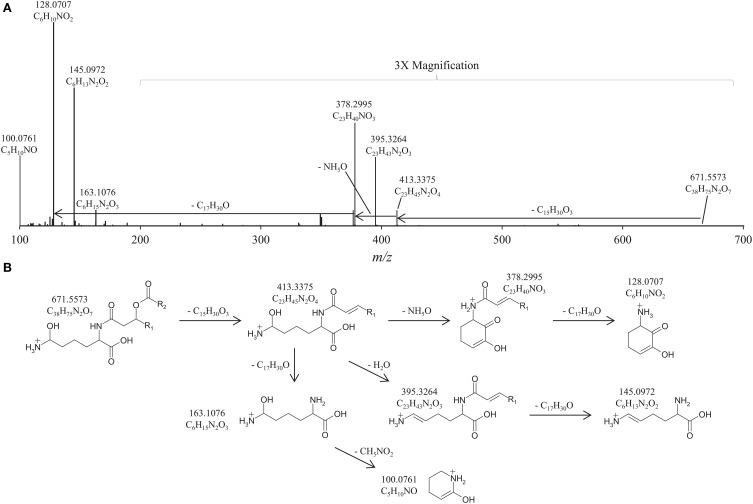
**(A)** High Resolution Accurate Mass/Mass Spectrometer (HRAM/MS) fragmentation of *Pseudopedobacter saltans* IPL *m/z* 671.5573 at retention time 35.97 min; **(B)** Proposed MS fragmentation of IPL with hydroxylysine head group (HLL). The relative abundance of the *m/z* 200–700 range is magnified 3×. The positive-ion ESI settings were as follows: capillary temperature, 275°C; sheath gas (N_2_) pressure, 35 arbitrary units (AU); auxiliary gas (N_2_) pressure, 10 AU; spray voltage, 4.0 kV; probe heater temperature, 300°C; S-lens, 50 V. Target lipids were analyzed with a mass range of *m/z* 400–1000 (resolution, 70,000), followed by data dependent MS^2^ (resolution, 17,500), in which the five most abundant masses in the mass spectrum were fragmented successively (normalized collision energy, 35; isolation width, 1.0).

Another pair of unknown IPLs (groups II and II') observed in the *P. saltans* lipid extract eluted at 29.2 and 34.9 min, respectively (Figure 1B). The lipids in both of these groups exhibited identical OL-like fragmentation with ESI/IT/MS MS^3^ products at *m/z* 129 and 145. As observed before for OL_HFA_ and LL_HFA_ compared to OLs and LLs, respectively, the later eluting group II' IPLs displayed MS^2^ fatty acid fragmentation losses *m/z* 16 larger than group II, indicating groups II and II' contain the same polar head group but group II' is potentially hydroxylated on one of the fatty acids chains. HPLC-HRAM/MS analysis confirmed the presence of hydroxylated fatty acids in group II', and revealed that in both groups II and II', fragmentation losses of fatty acids results in the *m/z* 163.1076 product, which has the exact elemental composition of protonated hydroxylysine, C_6_H_15_N_2_O_3_ (Figure 3). Further fragmentation (loss of H_2_O) results in the *m/z* 145.0972 product (C_6_H_13_N_2_O_2_). The loss of NH_5_O from the C_6_H_15_N_2_O_3_ head group results in the abundant proposed cyclic *m/z* 128.0707 product with elemental composition C_6_H_10_NO_2_, and the loss of CH_5_NO_2_ from the C_6_H_15_N_2_O_3_ head group results in the proposed cyclic *m/z* 100.0761 product (C_5_H_10_NO). These observed elemental compositions are similar to those for LL and LL_HFA_ but all with an additional oxygen (Table 1, Figure 3). We propose that these lipids contain hydroxylysine as the amino acid head group, which would explain the additional oxygen in the elemental composition, the observed fragments, and higher retention times (due to its more polar nature) compared to LL (Figure 1B). The fatty acid composition of the proposed hydroxylysine IPL (HLL) and fatty acid hydroxylated hydroxylysine lipids (HLL_HFA_) in groups II and II', respectively, are also the same as the OLs and LLs, including fatty acid hydroxylation (Table 1). The retention time of the amino acid released by acid hydrolysis from the isolated HLL_HFA_ (group II') was closer to the 5-hydroxylysine standard than any other amino acid standard, but the retention times were still 18 s apart (Table 3). It is therefore likely that the proposed hydroxylysine head group is hydroxylated in a different position than the hydroxylysine standard resulting in a slightly different retention time. This is in agreement with the study by Morin et al. (1998), which found different hydroxylation positions on lysine resulted in different amino acid analysis retention times.

### Biosynthetic pathway of lysine lipids

The synthesis pathway of LL has not yet been described but it appears to be linked to the OL synthesis. The first described synthetic pathway for OLs involves a *N*-acyltransferase coded by the *Ols*B gene transferring a 3-hydroxy acyl group from the constitutive acyl carrier protein (ACP) to the α-amino group of ornithine forming lyso-ornithine, and then an *O*-acyltransferase (encoded by the *Ols*A gene) transferring a fatty acyl chain from the acyl carrier protein to the 3-hydroxy group of the lyso-ornithine, forming OL (Weissenmayer et al., 2002; Gao et al., 2004). Vences-Guzmán et al. (2014) described the existence of a second pathway mediated by a bifunctional protein with two acyltransferase domains coded by the *Ols*F gene in some bacterial species, which have been described to form OL but lack the *Ols*AB genes. Vences-Guzmán et al. (2014) also described the formation of OL and LL, using MS analysis, in *E. coli* expressing the *Ols*F-coding protein from the *F. johnsoniae* strain UW101, but they did not confirm that LL were indeed produced by *F. johnsoniae*. However, our study confirms that *F. johnsoniae* synthesizes OL and LL. LLs have previously been reported in *Agrobacterium tumefaciens* by Tahara et al. (1976a) and in *Mycobacterium phlei* by Lerouge et al. (1988). The LL characterized in *M. phlei* is diacylglycerol based, while the structure of the *A. tumefaciens* LL is similar to the lysine lipids observed in this study by LC/MS and NMR, i.e., composed of an ester linked fatty acid moiety, and an amide linked β-OH-fatty acid.

Homologs of *Ols*F have been identified in genomes of γ-, δ-, and ε-Proteobacteria and in bacteria belonging to the CFB group (Vences-Guzmán et al., 2014). The genomes of *F. johnsoniae* and *Pedobacter heparinus* (both belonging to the CFB group) have been shown to harbor a homolog of *Ols*F (Vences-Guzmán et al., 2014). We searched for homologs of the *Ols*F-coding protein in genomes of *Pedobacter* species and *P. saltans*, whose lipid composition has been analyzed in our study. We detected putative homologs to *Ols*F in all *Pedobacter* genomes available (and in *P. saltans*) with an identity ≥70% to the *P. heparinus* putative *Ols*F (Vences-Guzmán et al., 2014), and an identity ≥40% to the *Ols*F protein of *F. johnsoniae*. These putative *Ols*F proteins in *Pedobacter* genomes have been previously annotated as hemolysines, as one of the genes of the hemolysin operon gene cluster, an acyltransferase catalyzing the transfer of a fatty-acyl group from acyl-ACP to α-amino groups of two lysine residues of the pro-toxin hemolysin converting it into a mature protein (Stanley et al., 1998). Considering this evidence, we speculate that the *Ols*F coding protein is involved not only in the synthesis of OL (Vences-Guzmán et al., 2014), but also in the synthesis of LL.

Additionally, in our study we report for the first time LLs that are modified by hydroxylation on one of its fatty acid chains and on the lysine head group. *P. saltans* produces a wide range of amino acid-containing IPLs including OL, OL_HFA_, LLs, LL_HFA_, HLL, and HLL_HFA_. All of the various ornithine and lysine lipids produced by *P. saltans* have the same fatty acid distribution (Table 1), suggesting a biosynthetic link. Hydroxylation of OL has been previously described and it is known to be mediated by the OL hydroxylase *Ols*C on ester-linked fatty acids (Rojas-Jimenez et al., 2005; Vences-Guzman et al., 2011), by the *Ols*D protein coding gene on the amide-linked fatty acid (González-Silva et al., 2011), or by the *Ols*E protein coding gene on the ornithine head group moiety (Rojas-Jimenez et al., 2005; Vences-Guzman et al., 2011). We performed protein blast searches for homologs of *Ols*C, *Ols*E of *Rhizobium tropici* (Rojas-Jimenez et al., 2005), and *Ols*D of *Burkholderia cenocepacia* but no putative homologs were detected in the currently available genomes of *Pedobacter* or *P. saltans*. However, we cannot rule out the presence of other putative OL hydroxylases in *Pedobacter* species genomes, which are expected to be quite divergent from the already described OL hydroxylases of *Rhizobium* and *Burkholderia* species, or that the hydroxylation of OL and LL in *Pedobacter* and *Pseudopedobacter* species is mediated by an alternative pathway.

### Environmental controls on lysine IPLs in *P. saltans*

**Figure 4:**
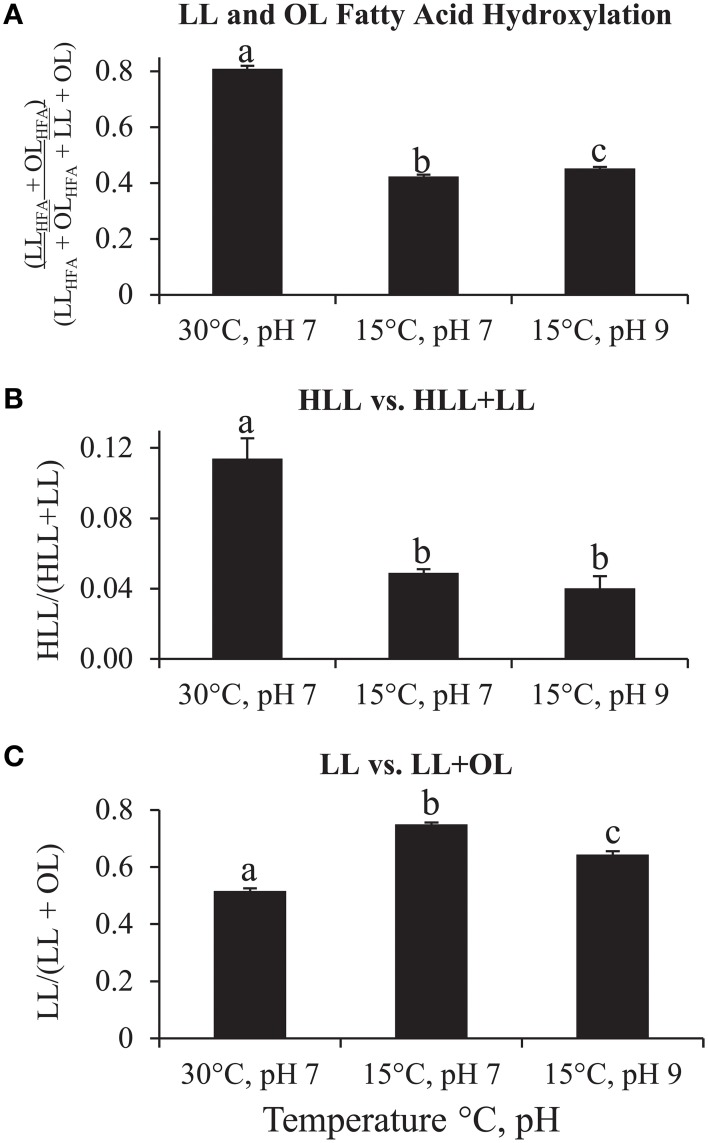
**Changes in amino acid-containing IPL abundance ratios based on HPLC-ESI/IT/MS chromatogram base peak area of each lipid group in** ***Pseudopedobacter saltans*** **lipid extract under 30°C/pH 7, 15°C/pH 7, and 15°C/pH 9 growth conditions. (A)** Ratio of total fatty acid hydroxylated lysine and ornithine lipids (LL_HFA_ + OL_HFA_) to total hydroxylated plus total unhydroxylated fatty acid lysine and ornithine lipids (LL_HFA_ + OL_HFA_ + LL + OL); **(B)** Ratio of total hydroxylysine lipids (HLL) to total HLL plus total LL; **(C)** Ratio of total LL to total LL plus total OL. Student's *t*-test statistically significant differences (*p* < 0.05) of IPL ratios between different growth conditions are represented by letters (a, b, c) over each bar (different letters represent statistically significant differences between bar graph values within each graph, values with the same letter within each graph are not statistically different).

The novel characterization of HLL, and the high abundance of LL_HFA_ and OL_HFA_ in the lipid extract of *P. saltans* raises the question of their functional significance. Various studies have shown that certain bacteria increase fatty acid hydroxylation of OLs under temperature and pH stress (Taylor et al., 1998; Rojas-Jimenez et al., 2005; Vences-Guzman et al., 2011). The high abundance of LL_HFA_ and OL_HFA_ in *P. saltans* suggested that these lipids could be associated with in environmental stress response. We tested this hypothesis for LLs and OLs in *P. saltans* by growing triplicate cultures under three temperature/pH combinations: 30°C/pH 7, 15°C/pH 7, 15°C/pH 9. The ratios of LL and OL abundance, and the degree of fatty acid hydroxylation in *P. saltans* changed under different temperature and pH conditions (Figure 4). There was a statistically significant increase in the ratio of the sum of LL_HFA_ and OL_HFA_ (LL_HFA_ + OL_HFA_) to total LLs and OLs (LL_HFA_ + OL_HFA_ + LL + OL) at higher temperatures and higher pH, with the largest ratio increase due to temperature (*p* < 0.0001) and a smaller increase due to pH rise (*p* = 0.0085, Figure 4A). There was also a significant increase in the ratio of HLL to the sum of HLL and LL at higher temperatures (*p* = 0.0115, Figure 4B). There were significant decreases in the ratio of total LLs vs. the sum of total LLs and OLs at higher temperatures (*p* < 0.0001) and higher pH (*p* = 0.0025), with the largest LL/(LL + OL) ratio decrease also due to temperature and a moderate decrease due to pH (Figure 4C).

There appears to be an OL and LL fatty acid hydroxylation stress response to temperature and pH in *P. saltans*, similar to the fatty acid hydroxylation stress response to temperature and pH observed in various other bacteria, which is proposed to increase membrane hydrogen bonding and stability (Taylor et al., 1998; Rojas-Jimenez et al., 2005; Vences-Guzman et al., 2011). In this case, there is a greater response to temperature than pH for *P. saltans*. The lysine head group hydroxylation also follows the same trend as fatty acid hydroxylation with more hydroxylation at higher temperature, as also observed in OL head group hydroxylation in *R. tropici* (Rojas-Jimenez et al., 2005; Vences-Guzman et al., 2011). OLs may also be preferentially produced over LLs at higher temperatures or pH levels in order to provide membrane stability due to the higher water solubility of ornithine than lysine. The various IPL composition changes due to temperature and pH show that the cell membrane of *P. saltans* is very dynamic in response to changing environmental conditions.

## Conclusions

This is the first study to identify LL fatty acid hydroxylation and LL head group hydroxylation in membrane lipids. Confirmation of OL and LL formation in *F. johnsoniae* and *P. saltans* and the presence of *Ols*F putative homologs in *P. saltans* suggest the *Ols*F gene coding protein is possibly involved in OL and LL biosynthesis in both species, however, potential pathways of OL and LL hydroxylation in *P. saltans* are still undetermined.

LL and OL fatty acid hydroxylation and LL vs. OL fractional abundance changes due to temperature and pH in microbial culture experiments were observed, thus expanding knowledge of microbial response to environmental change. The IPLs of related soil bacteria (i.e., *Pedobacter* sp.) should be characterized as well to investigate stress biomarker potential of environmental conditions such as temperature or pH. Analysis of soil samples is needed as well to reveal if these membrane lipid responses to temperature and pH changes can be observed in the environment.

### Conflict of interest statement

The authors declare that the research was conducted in the absence of any commercial or financial relationships that could be construed as a potential conflict of interest.

## Footnotes

^1^Commission Regulation (EC) no., 152/2009 of January 27 2009. Laying down the methods of sampling and analysis for the official control of feed. Official Journal of the European Union L 54/28 Part F.

^2^European Pharmacopoeia chapter 2.2.56, USP chapter 1052 (2005) Method 1 for Analysis.
