# Supplementary material for: Lysine and novel hydroxylysine lipids in soil bacteria: amino acid membrane lipid response to temperature and pH in Pseudopedobacter saltans
Source: Front Microbiol. 2015 Jun 29;6:637. doi: 10.3389/fmicb.2015.00637 (PMC4484230; doi:10.3389/fmicb.2015.00637)
Supplement: Supplementary file 1 [file Image1.PDF]

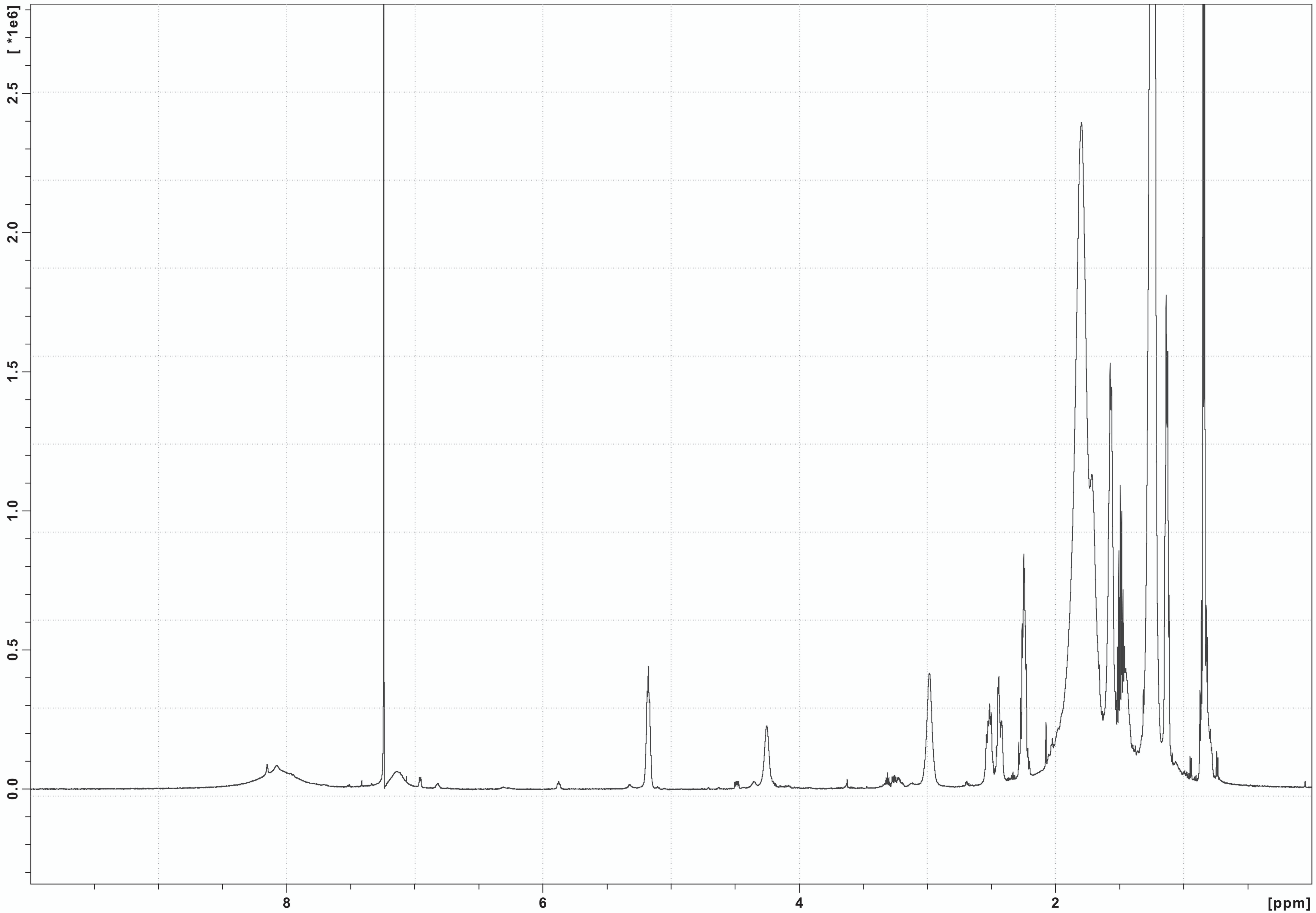

Figure S1: ^{1}H nuclear magnetic resonance (NMR) spectra of lysine lipid (LL).

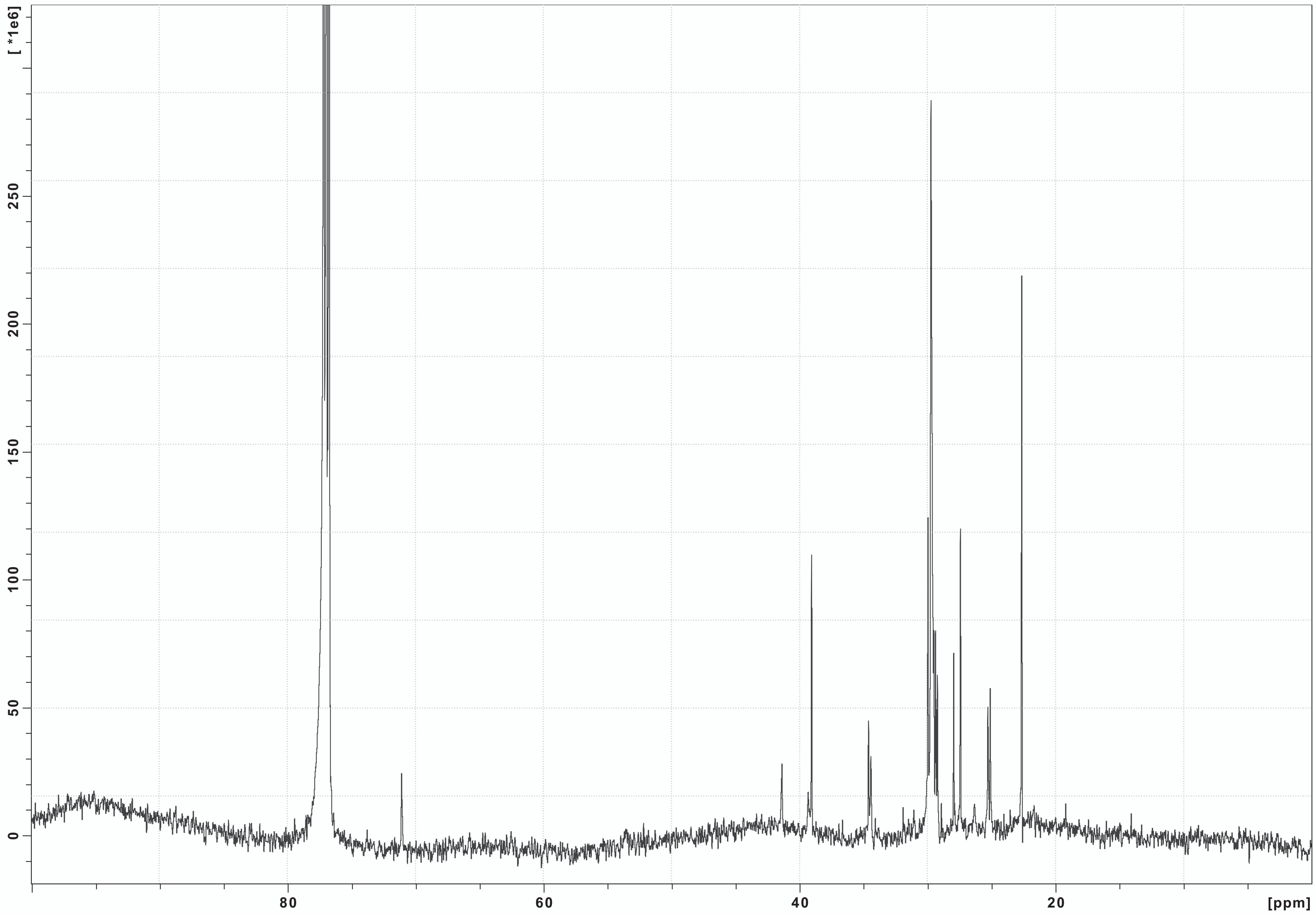

Figure S2: ^{13}C nuclear magnetic resonance (NMR) spectra of lysine lipid (LL).

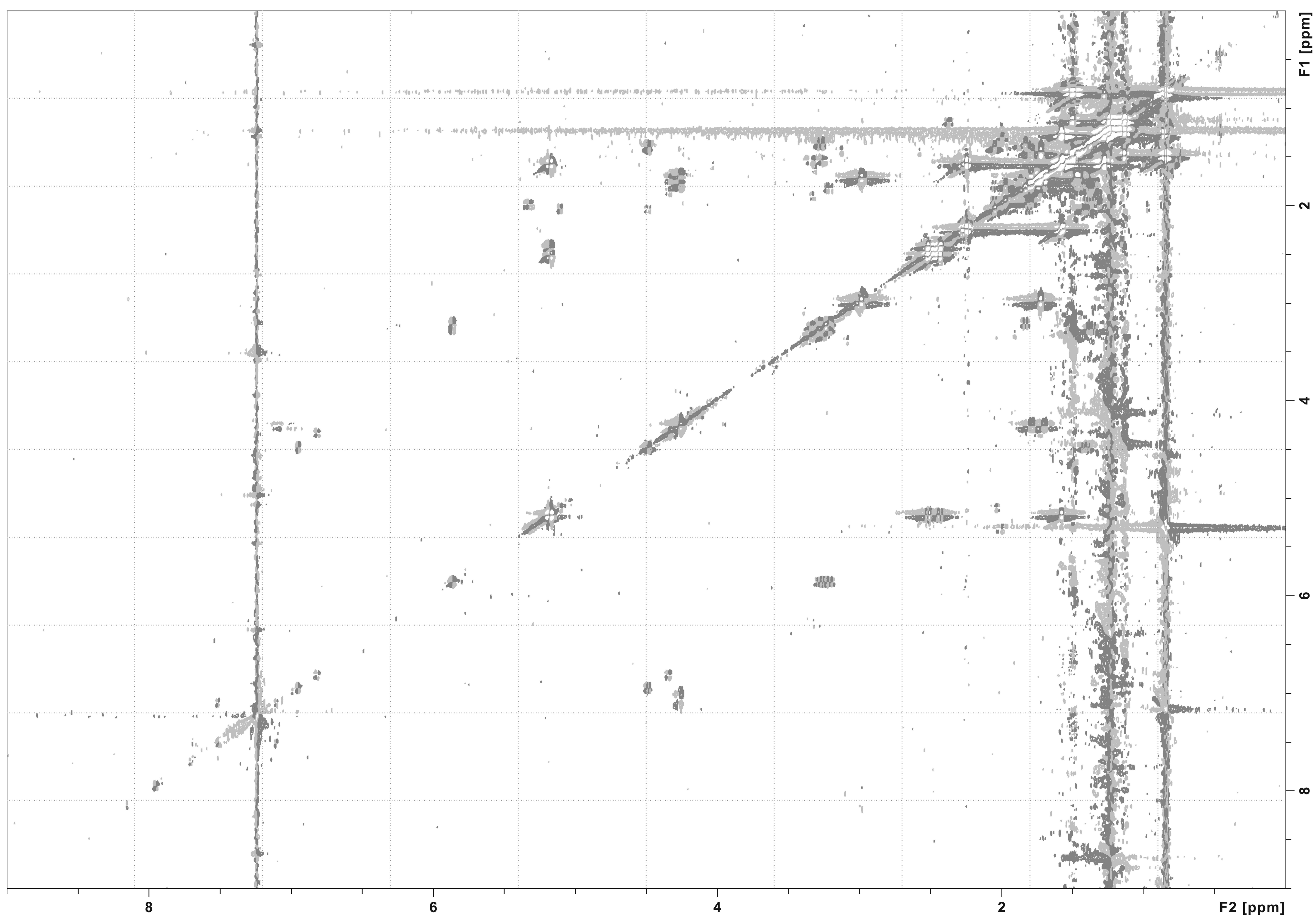

Figure S3: Correlation spectroscopy (COSY) two dimensional (2D) nuclear magnetic resonance (NMR) spectra of lysine lipid (LL).

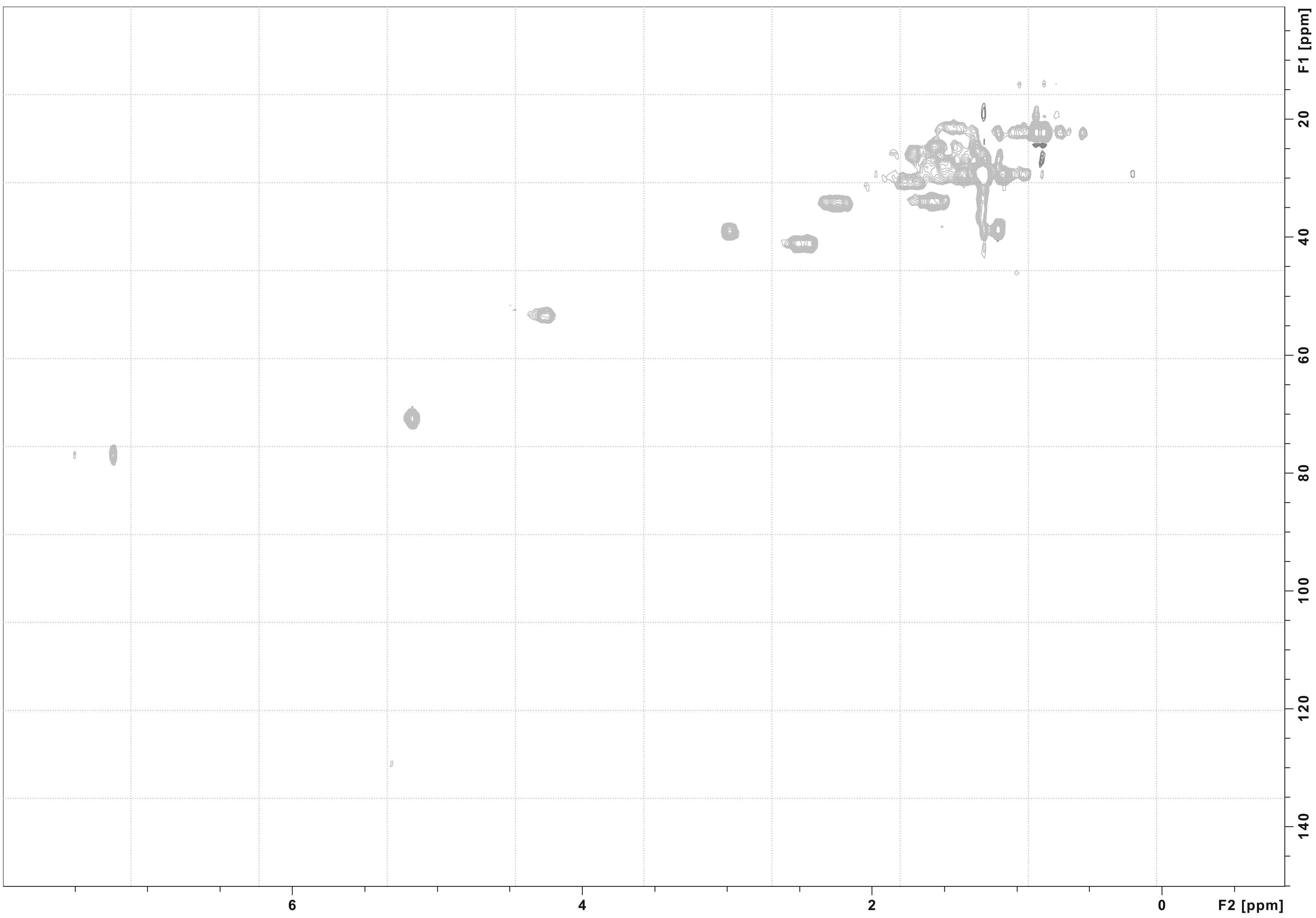

Figure S4: Heteronuclear single-quantum correlation spectroscopy (HSQC) two dimensional (2D) nuclear magnetic resonance (NMR) spectra of lysine lipid (LL).

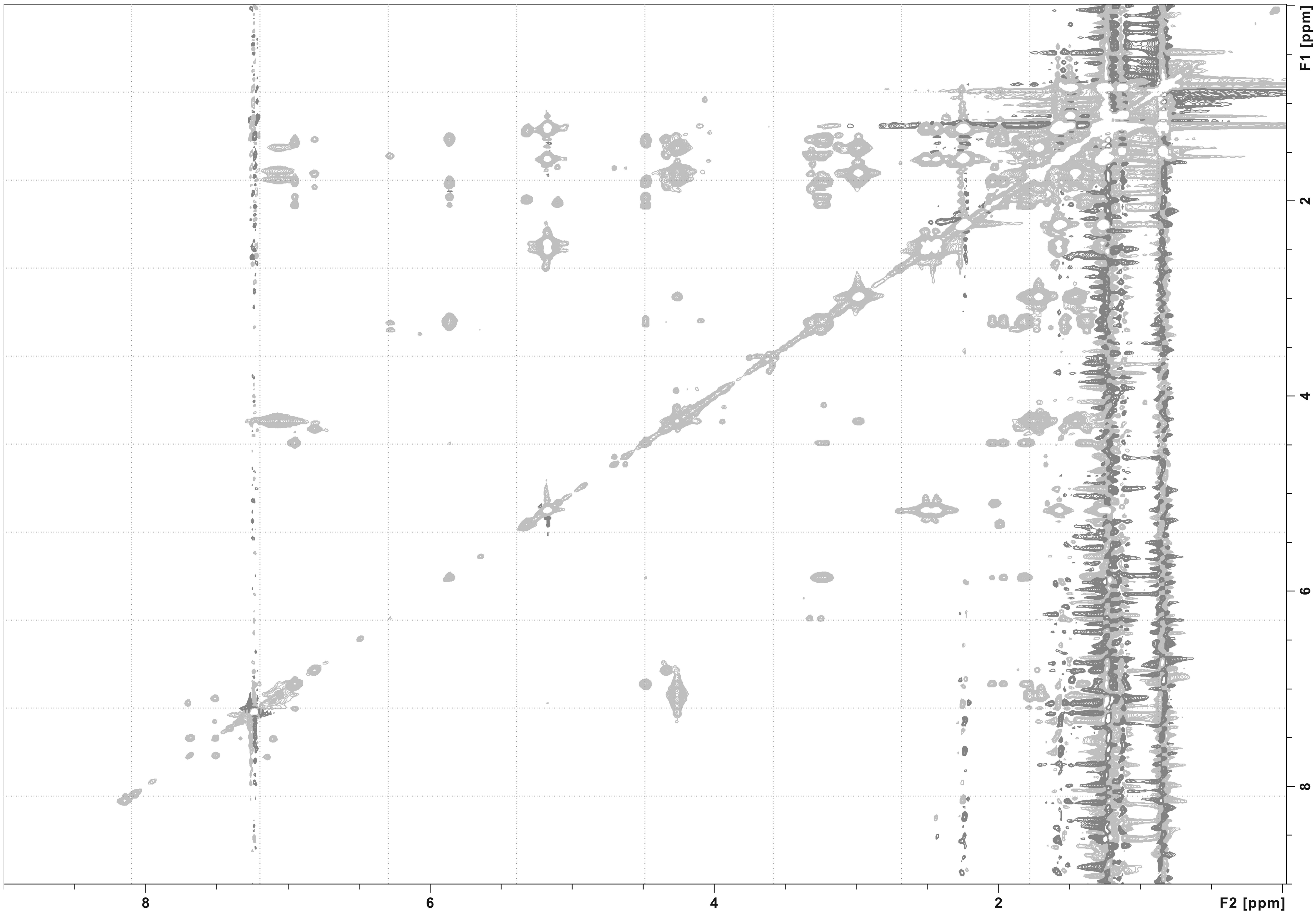

Figure S5: Total correlation spectroscopy (TOCSY) two dimensional (2D) nuclear magnetic resonance (NMR) spectra of lysine lipid (LL).

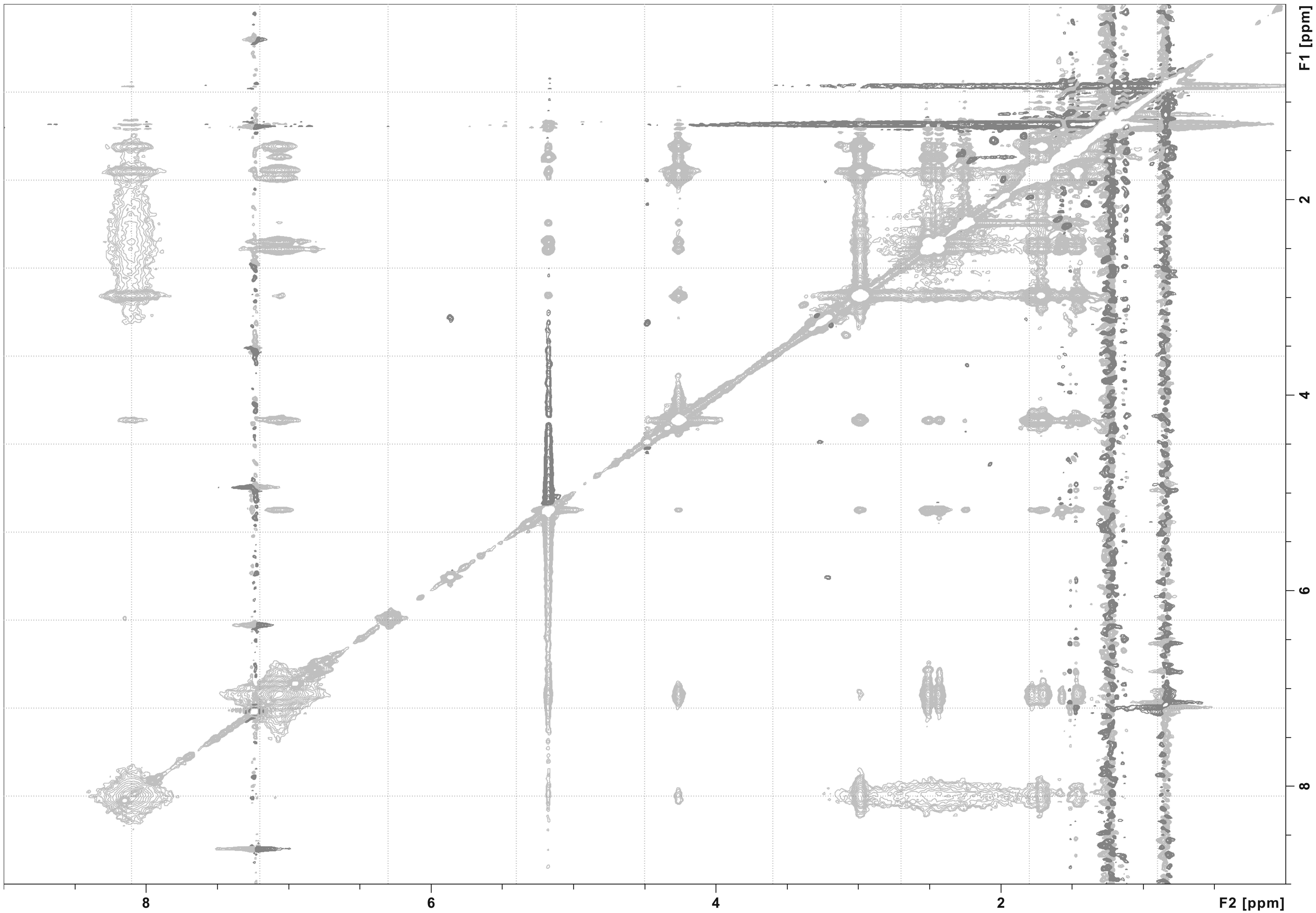

Figure S6: Nuclear Overhauser effect spectroscopy (NOESY) two dimensional (2D) nuclear magnetic resonance (NMR) spectra of lysine lipid (LL).

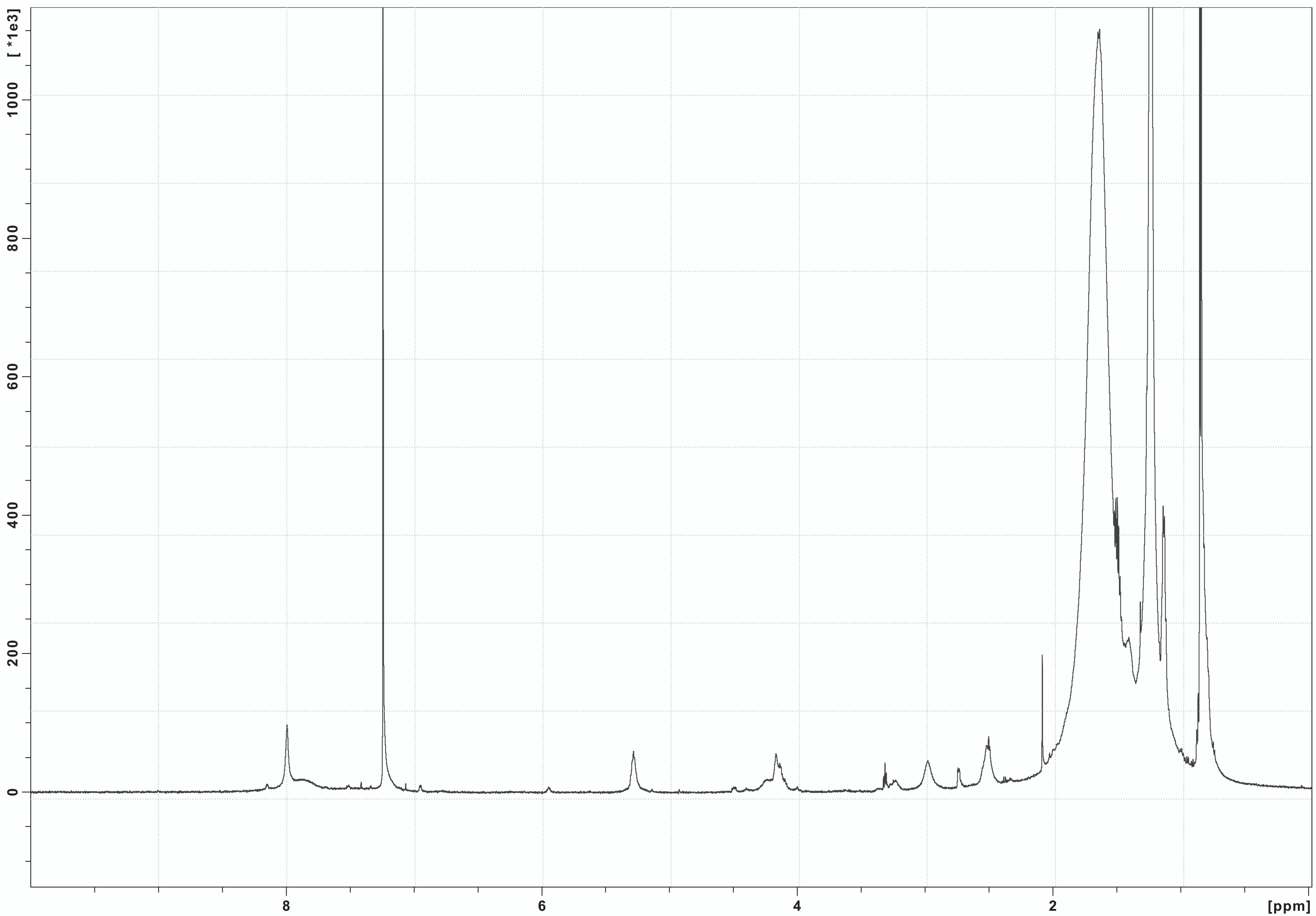

Figure S7: ^{1}H nuclear magnetic resonance (NMR) spectra of fatty acid hydroxylated lysine lipid (LL_{HFA}).

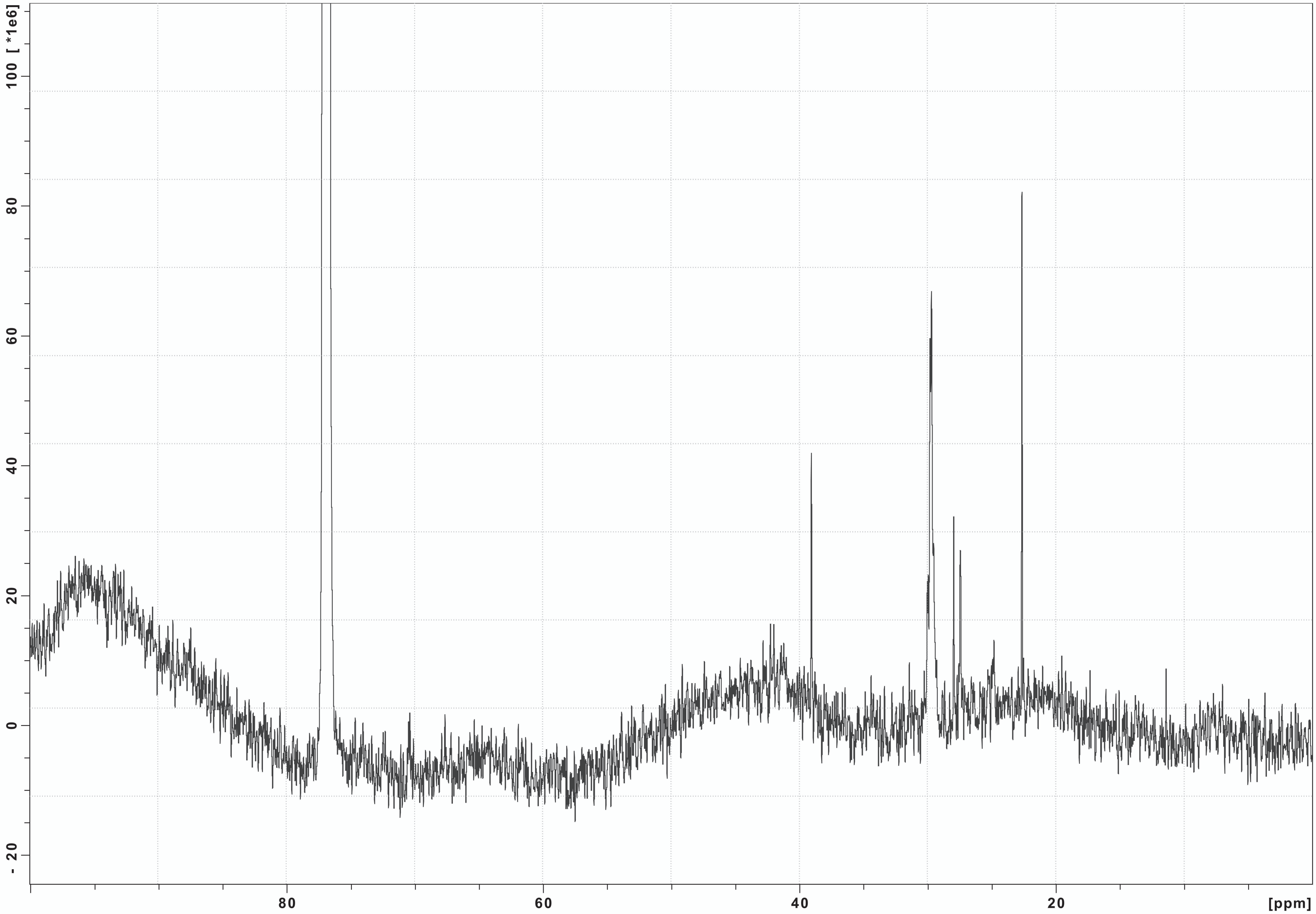

Figure S8: ^{13}C nuclear magnetic resonance (NMR) spectra of fatty acid hydroxylated lysine lipid (LL_{HFA}).

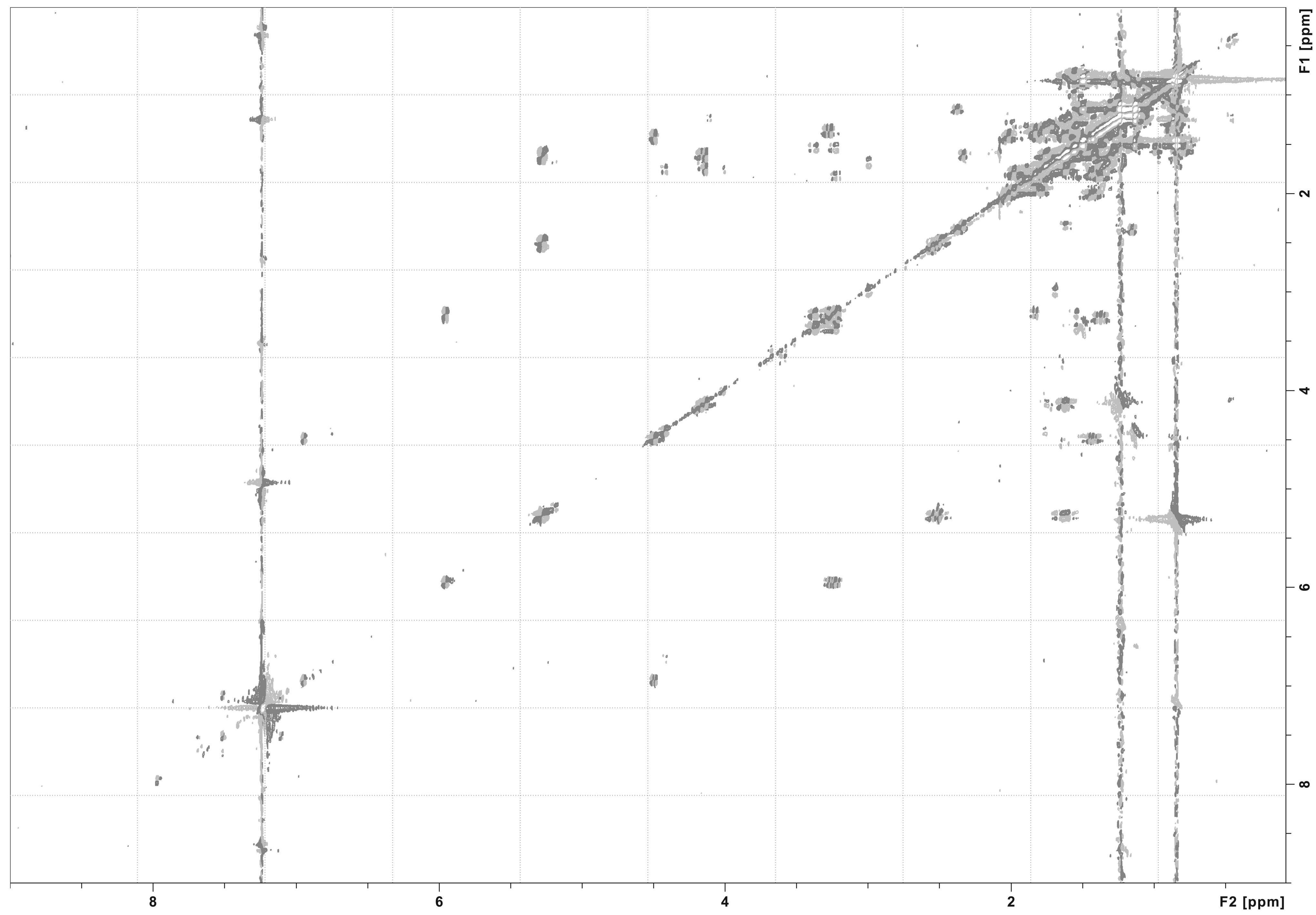

Figure S9: Correlation spectroscopy (COSY) two dimensional (2D) nuclear magnetic resonance (NMR) spectra of fatty acid hydroxylated lysine lipid (LL_{HFA}).

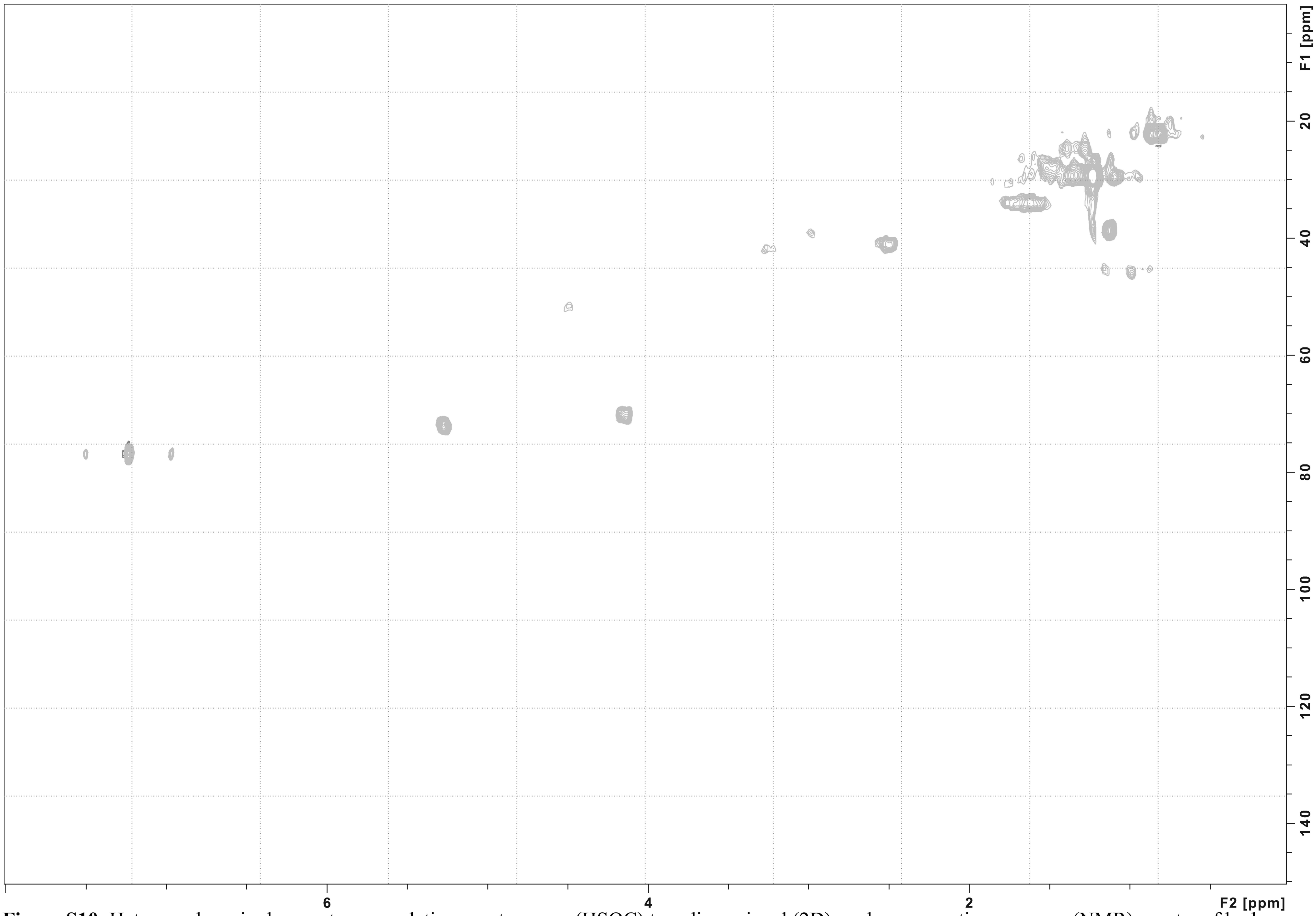

Figure S10: Heteronuclear single-quantum correlation spectroscopy (HSQC) two dimensional (2D) nuclear magnetic resonance (NMR) spectra of hydroxylated fatty acid lysine lipid (LL_{HFA}).

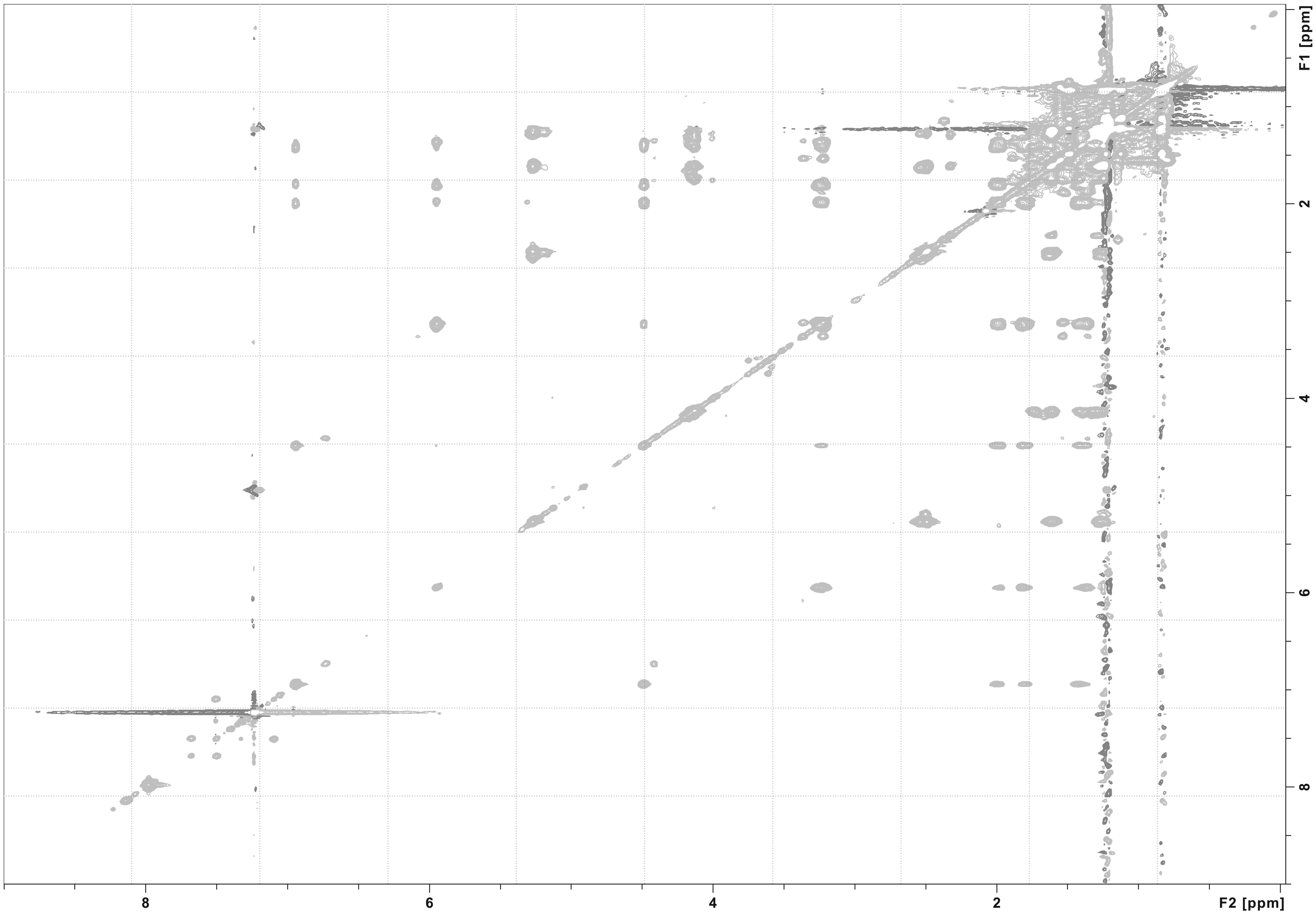

Figure S11: Total correlation spectroscopy (TOCSY) two dimensional (2D) nuclear magnetic resonance (NMR) spectra of fatty acid hydroxylated lysine lipid (LL_{HFA}).

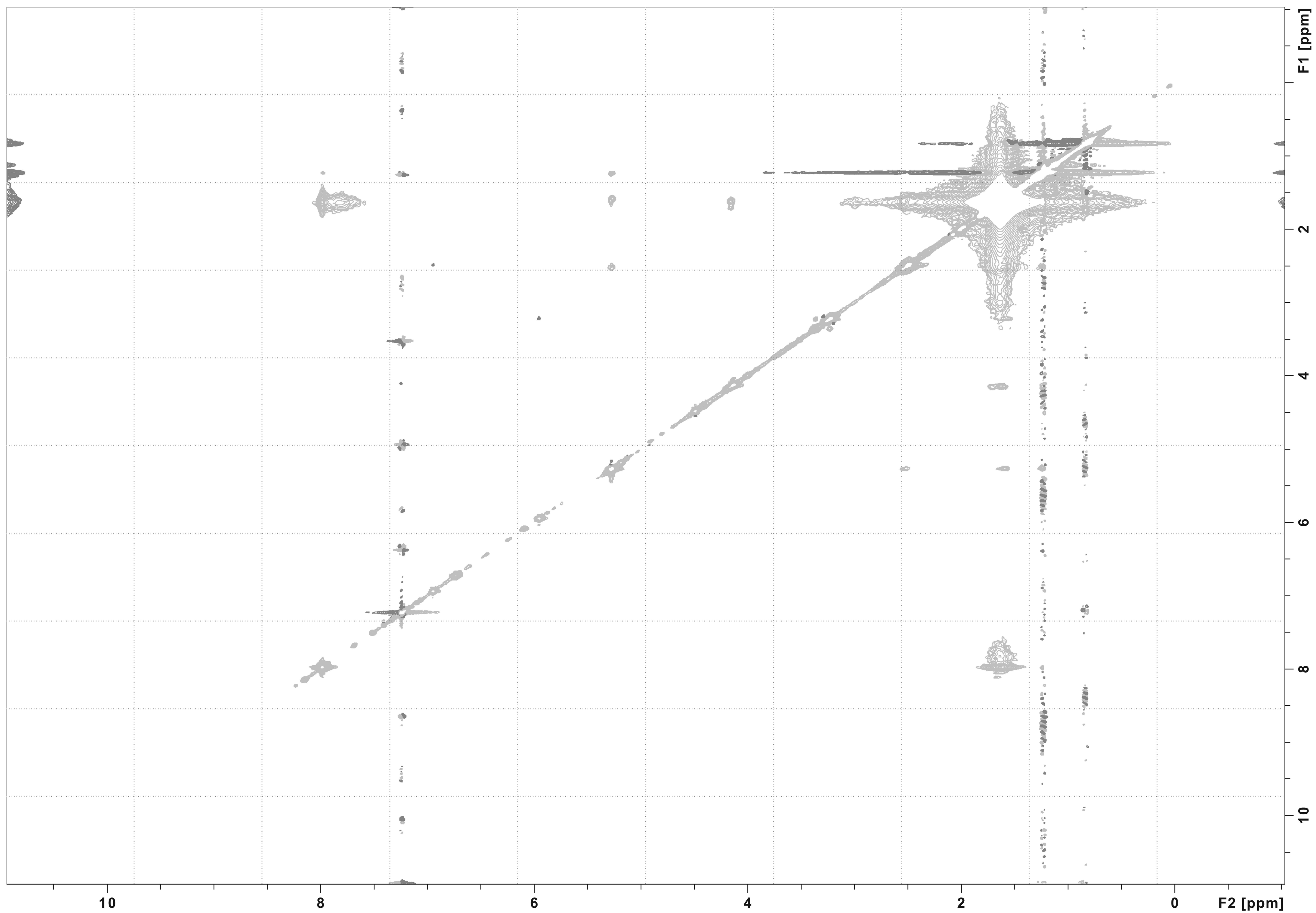

Figure S12: Nuclear Overhauser effect spectroscopy (NOESY) two dimensional (2D) nuclear magnetic resonance (NMR) spectra of fatty acid hydroxylated lysine lipid (LL_{HFA}).
